# Massachusetts Veterinary Association

**Published:** 1898-01

**Authors:** Henry S. Lewis

**Affiliations:** Secretary


Secretary.

### MASSACHUSETTS VETERINARY ASSOCIATION.

THE regular monthly meeting was held at 19 Boylston Place, on Wednesday evening, October 27th. President Winchester called the meeting to order at 8 o'clock. Members present: Drs. Lee, Lewis, McLaughlin, Parker, Pierce, Soule, Stickney, Williams, Winchester, Winslow. Minutes of previous meeting were read and approved. Drs. Albert A. Etienne and Clarence E. Burchsted were elected members of the Association.

Drs. Soule, McLaughlin, Winslow, and Pierce reported interesting cases.

The subject of a State veterinary law was fully discussed, and a committee was appointed to draft a bill and report at the December meeting.

HENRY S. LEWIS,
Secretary.

